# Characterization of Experimentally Observed Complex Interplay between Pulse Duration, Electrical Field Strength, and Cell Orientation on Electroporation Outcome Using a Time-Dependent Nonlinear Numerical Model

**DOI:** 10.3390/biom13050727

**Published:** 2023-04-23

**Authors:** Maria Scuderi, Janja Dermol-Černe, Tina Batista Napotnik, Sebastien Chaigne, Olivier Bernus, David Benoist, Daniel C. Sigg, Lea Rems, Damijan Miklavčič

**Affiliations:** 1 Faculty of Electrical Engineering, University of Ljubljana, SI-1000 Ljubljana, Slovenia; 2 INSERM, CRCTB, U 1045, IHU Liryc, University of Bordeaux, F-33000 Bordeaux, France; 3 Medtronic, Cardiac Ablation Solutions, Minneapolis, MN 55105, USA

**Keywords:** finite element model, time domain, electroporation, pulsed-field ablation, cardiomyocyte, intracellular calcium, lethal electric field strength, cell orientation, anisotropy

## Abstract

Electroporation is a biophysical phenomenon involving an increase in cell membrane permeability to molecules after a high-pulsed electric field is applied to the tissue. Currently, electroporation is being developed for non-thermal ablation of cardiac tissue to treat arrhythmias. Cardiomyocytes have been shown to be more affected by electroporation when oriented with their long axis parallel to the applied electric field. However, recent studies demonstrate that the preferentially affected orientation depends on the pulse parameters. To gain better insight into the influence of cell orientation on electroporation with different pulse parameters, we developed a time-dependent nonlinear numerical model where we calculated the induced transmembrane voltage and pores creation in the membrane due to electroporation. The numerical results show that the onset of electroporation is observed at lower electric field strengths for cells oriented parallel to the electric field for pulse durations ≥10 µs, and cells oriented perpendicular for pulse durations ~100 ns. For pulses of ~1 µs duration, electroporation is not very sensitive to cell orientation. Interestingly, as the electric field strength increases beyond the onset of electroporation, perpendicular cells become more affected irrespective of pulse duration. The results obtained using the developed time-dependent nonlinear model are corroborated by in vitro experimental measurements. Our study will contribute to the process of further development and optimization of pulsed-field ablation and gene therapy in cardiac treatments.

## 1. Introduction

Electroporation is the underlying mechanism in a new promising cardiac ablation method—Pulsed Field Ablation (PFA)—currently being developed for the treatment of atrial fibrillation and other cardiac arrhythmias [1,2,3]. Electroporation has also been attributed to performing a role in cardiac defibrillation [4] and has shown promise for cardiac regeneration based on gene therapy [5,6].

Electroporation is a phenomenon where the application of high-voltage electric pulses to isolated cells or tissues transiently increases membrane permeability allowing the transport of ions and molecules otherwise deprived of or having hindered transmembrane transport mechanisms [7,8]. Following electroporation, cells may recover and survive (i.e., reversible electroporation), or lose homeostasis and undergo cell death (i.e., irreversible electroporation). The increase in cell membrane permeability is due to a supraphysiological transmembrane voltage (more than a few 100 mV) induced by an external electric field [9,10] and can involve pore formation in the lipid bilayer of the cell membrane, oxidative lipid damage, and structural alteration of specific membrane proteins [11]. The induced transmembrane voltage varies with the position on the membrane and depends on cell size, shape, and orientation with respect to the applied electric field [11,12,13]. The time course of the induced transmembrane voltage for spherical and other regularly shaped cells can be determined analytically, and for cells with more complex and irregular shapes, such as isolated cardiomyocytes, it can be calculated numerically [12,13]. In general, electroporation follows a “size rule” in the sense that larger cells are electroporated at lower electric field strengths than smaller ones, as the induced transmembrane voltage is proportional to the electric field strength and cell radius. 

Cardiac tissue is composed of different types of cells (e.g., cardiomyocytes, fibroblasts, smooth muscle cells, immune cells, neuronal cells, etc. [14]), whereby the target of the electroporation therapy are typically cardiomyocytes. Cardiomyocytes are cardiac muscle cells that are elongated and rod-shaped with a non-smooth membrane surface and a complex array of tubules. Cardiac transverse tubules (t-tubules) are invaginations of cardiomyocyte sarcolemma and are involved in the maintenance of resting membrane voltage, action potential initiation, regulation and propagation, signaling transduction, and the coupling for the excitation-contraction cycle [15]. Cardiac myocytes are organized in fibers and their orientation within the heart is variable. For example, in ventricular tissue, fibers turn from a circumferential orientation on the epicardium to an apicobasal orientation in the endocardium [16,17,18]. Fiber orientation in the atria is more irregular than that of the ventricles [19,20,21]. In the context of cardiac arrhythmia treatment, a wide range of pulse shapes and durations have been investigated and used in vitro and in vivo, from exponentially decaying pulses to monophasic pulses with duration ranging from milliseconds to nanoseconds, and most recently, short µs-long biphasic pulses referred to as high-frequency irreversible electroporation (HFIRE) [22,23,24,25,26,27,28,29]. The orientation of elongated cardiac cells and the different pulse parameters (e.g., amplitude, duration, number, and repetition frequency), which are used for the treatment, might affect the efficiency of PFA treatment. An efficient way to determine the effect of an external electric field on a cell is by numerical modeling combined with in vitro experimental results.

Milan et al. [30] studied the induced transmembrane voltage due to the applied external electric field on a single cardiomyocyte from the modeling point of view and showed that the shape of cardiomyocytes can be approximated by a prolate spheroid. Under steady-state conditions, the cardiomyocytes (and other elongated cells) are predicted to be more affected when oriented parallel to an external electric field compared to perpendicular orientation. However, it was observed experimentally that cardiomyocytes are electroporated by nanosecond pulses at lower electric field strengths when oriented perpendicularly to the electric field compared to the parallel orientation [27,31]. Surprisingly, to some extent, effects associated with electroporation do not always follow the “size rule” [32] as intuitively expected based on simple steady-state calculations. A study published by Dermol-Černe et al. [31] showed that parallel orientation is more affected than perpendicular orientation when a single monophasic pulse of >1 µs duration is applied to different electric fields. Chaigne et al. [33] recently published a study in which authors investigated the lethal electric field strengths of a single-oriented cardiomyocyte when applying a single monophasic pulse of 10 ms or 100 μs pulse duration. The authors showed that the cells oriented perpendicular to the electric field had a lower lethal threshold at 100 µs than parallel cells, but cells oriented parallel to the electric field had a lower lethal threshold at 10 ms than perpendicular cells [33]. Interestingly, they showed that perpendicular orientation is more affected than the parallel one when using 100 μs pulse duration which seems to be in contrast to the findings from Dermol-Černe et al. [31]. To gain a better mechanistic understanding of the in vitro experimental data published by Dermol-Černe et al. [31] and Chaigne et al. [33], we developed a time-dependent nonlinear numerical model which included membrane electroporation. We built upon a previously published steady-state model of a single cardiomyocyte with realistic shape and simplified prolate spheroid shape, which was exposed to electric field [30]. In contrast to the steady-state model, our model also captures the nonlinear behavior of the induced transmembrane voltage as the membrane is being electroporated [34,35]. We used our developed model to investigate the effect of cardiomyocyte orientation on electroporation induced by a single monophasic pulse of durations from 10 ms to 100 ns pulses. These pulse parameters were chosen specifically to be able to compare the modeling findings with in vitro experimental data previously reported by Dermol-Černe et al. [31] and Chaigne et al. [33]. Since the cardiomyocyte model did not include t-tubules, we have numerically modeled these by varying the membrane capacitance to determine how these would affect the induced transmembrane voltage and electroporation. The modeling results suggest that the orientation at which cardiomyocytes become preferentially affected by electroporation pulses depends in a complex way both on pulse duration and the electric field strength. The model findings corroborate and bring better mechanistic understanding on the apparently conflicting experimental results published previously [31,33]. In addition, the modeling results may become relevant for interpreting results at the tissue level in clinical applications of PFA.

## 2. Materials and Methods

### 2.1. The Time-Dependent Numerical Model with Electroporation

**Figure 1:**
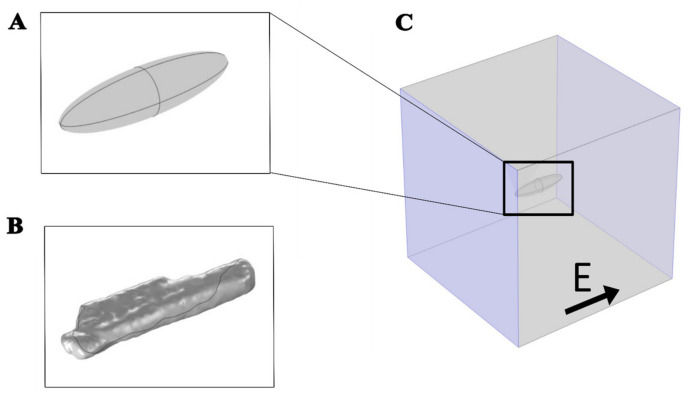
Geometries used to represent a cardiomyocyte with an electric field applied parallel to the long axis of the cell. (**A**) Prolate spheroid geometry, 120 µm long, 30 µm wide, and 30 µm high. (**B**) Real-shaped geometry 142 µm long, 36 µm wide, and 21 µm high. Both the real-shaped geometry and its prolate spheroid approximation were the same as in Milan et al. [30]. (**C**) The cell was at the center of the box when the electric field was applied parallel to the long axis of the cell. The violet-colored sides of the box represent the electrodes to which the voltage was applied.

A time-dependent nonlinear numerical model was developed, including the phenomenon of electroporation, to study the effect of the external applied electric field on a cardiomyocyte. The model was developed using COMSOL Multiphysics 5.6 software (Comsol, Inc., Burlington, MA, USA). Two different geometries, prolate spheroid (Figure 1A) and the real-shaped geometry of a cardiomyocyte (Figure 1B) were used to represent a single cardiomyocyte. The real-shaped geometry of a cardiomyocyte was kindly provided by Milan et al. [30]. Both geometries were modeled in the center of the simulation cube with dimensions 400 μm × 400 μm × 400 μm (Figure 1C). 

The electric potential distribution, V, in the intracellular and extracellular subdomains was calculated in the *AC/DC module*, *Electric Currents* physics by solving the Laplace equation:

(1) $$▽\cdot \left[\left(\sigma_{i,e}+\varepsilon_{i,e}\frac{\partial }{\partial t}\right)▽V_{i,e}\right]=0$$

where $\sigma_{i,e}$ and $\varepsilon_{i,e}$ denote, respectively, the conductivity and the dielectric permittivity of either intracellular (subscript *i*) or extracellular (subscript *e*) medium. The cell membrane was modeled using the *Contact Impedance Boundary Condition:*

(2) $$n\cdot J=\frac{1}{d_{m}}\left(\sigma_{m}+\varepsilon_{0}\varepsilon_{m}\frac{\partial }{\partial t}\right)\left(V_{i}-V_{e}\right)$$

where ***n*** is the normal vector, ***J*** is the current density, $d_{m}$ is the cell membrane thickness, $\sigma_{m}$ is the cell membrane conductivity, $\varepsilon_{m}$ is the cell membrane permittivity, $\varepsilon_{0}$ is the permittivity of the vacuum, and *V_e_* and *V_i_* are the electric potentials at the outer and inner surfaces of the membrane, respectively. The induced transmembrane voltage (TMV) is calculated as the difference between the extracellular, *V_e_*, and intracellular, *V_i_*, electric potential, TMV = *V_i_* − *V_e_*.

A monophasic electric pulse with different pulse durations of 10 ms, 1 ms, 100 µs, 10 µs, 1 µs, and 100 ns was applied on the two opposite boundaries of the simulation cube, either parallel or perpendicular to the main axis of the cardiomyocyte. The remaining four faces of the cube were modeled as insulating surfaces. The electric pulse was obtained by subtracting two Heaviside functions using the COMSOL’s built-in function *flc1hs* [36]. The pulse rise time was set to 1/100 of the pulse duration. The values of the electric field applied in the numerical model were from 10 V/cm to 10^5^ V/cm.

Pore formation was calculated as a function of time and thus can only be added in the time-dependent simulations. The pore formation was described by the following differential Equation (3) implemented in COMSOL Multiphysics as a *Weak Form Boundary partial differential equation:*

(3) $$\frac{dN}{dt}=\alpha e^{{\left(\frac{U_{m}}{V_{ep}}\right)}^{2}}-\alpha \frac{N}{N_{0}}{e^{\left(-q\right)}}^{{\left(\frac{U_{m}}{V_{ep}}\right)}^{2}}$$

where *N* represents the pore density (number of pores per unit area), *U_m_* denotes the TMV, *N*_0_ is the pore density when *U_m_* = 0 V, and *V_ep_*, *a,* and *q* are model parameters, respectively. The first term of Equation (3) represents pore creation and the second one the pore annihilation [37,38].

The increase in cell membrane conductivity during electroporation, *σ_ep_*, was calculated as:

(4) $$\sigma_{ep}=\sigma_{m}+N\frac{2\pi {r_{p}}^{2}\sigma_{p}d_{m}}{\pi r_{p}+2d_{m}}$$

where *r_p_* and *σ_p_* are the radius and conductivity of a single pore, respectively. The first term of Equation (4) represents the passive membrane conductivity and the second one is the increase in conductivity due to electroporation [37,38]. The parameters used in the numerical model are shown in Table 1.

### 2.2. In Vitro Experiments: Calcium Transients in Cardiomyocyte-Derived Cell Lines 

We compared our numerical model with experimental results previously published by Dermol-Černe et al. [31]. The electroporation extent was evaluated by calcium uptake to cells from the external medium [41]. In brief, H9c2 rat cardiac myoblast cell line (European Collection of Authenticated Cell Cultures ECACC 88092904) and AC16 human cardiomyocyte cell line (Merck Millipore, SCC109) were stained with a fluorescent calcium indicator Fura-2 AM and exposed to a single monophasic electric pulse of different durations, ranging from 10 ms to 100 ns. In each experiment, cultured cells were exposed to a single monophasic pulse of the same duration, but increasing voltage was applied 8 to 12 min apart that allow the cells to reseal and restore low internal calcium concentration. Cells were monitored under a fluorescence microscope (Zeiss Axiovert 200, Oberkochen, Germany) in ratiometric measurements using two excitation wavelengths (340 and 380 nm). When internal calcium concentration increased due to calcium uptake, the Fura-2 340/380 ratio increased. With the use of an image-processing program ImageJ (National Institutes of Health, Bethesda, MD, USA), the orientations of cells in an electric field were determined and a mean ratio of Fura-2 340/380 was calculated for each cell. The Fura-2 signal was expressed as a Fura-2 ratio 340/380 peak change, which occurred 8 s after the pulse application (see Supplementary Figure S1). The in vitro data of the aforementioned experimental work are presented in a way that it is possible to evaluate two different outcomes. The first outcome is the quantification of the difference in fluorescent calcium indicator Fura-2 signal in parallel and perpendicular cells already published in Dermol-Černe et al. [31]. The second outcome is the determination of the Fura-2 ratio 340/380 peak change observed when the cells are oriented parallel or perpendicular to the applied electric field (unpublished data and presented in this study). Statistical analysis for the aforementioned cell experiments was performed using Excel and SigmaPlot 11.0 (Systat Software, Chicago, IL, USA). The results in Figure 6 (unpublished data) are expressed as means ± SD. The normality of the data distribution was tested with the Kolmogorov–Smirnov test. Significant differences (*p* < 0.05) in Fura-2 responses were determined by paired *t*-test. In very rare occasions (2 out of 60 groups), the distribution was not normal, and instead, the Wilcoxon signed-rank test was used; however, in these two cases, the differences were not significant. 

### 2.3. In Vitro Experiments: Electric Field Effect on Diastolic Calcium Level in Primary Cardiomyocytes 

A detailed methodology was provided before [33]. Briefly, adult rat cardiomyocytes were enzymatically isolated from the left ventricle and loaded with 4 µM Fura-2 AM (Invitrogen) to monitor intracellular calcium. Myocytes were placed between parallel electrodes with 4 mm gap distance and exposed to monophasic 100 µs pulses of increasing voltage: 80 V, 140 V, and lethal high voltage pulses. For the latter, the voltage applied was different for cells oriented parallel and perpendicular relative to the electric field as a consequence of their different sensitivity to pulsed electric fields. The diastolic calcium level, corresponding to the 340/380 ratio at rest, was measured prior to and at the maximum level following the application of the electroporating pulse. Statistical analysis for this experiment was performed with a two-way repeated-measure ANOVA, followed by a Bonferroni multiple comparison test using SigmaPlot 14.0. 

## 3. Results and Discussion

### 3.1. The Time-Dependent Numerical Model with Included Electroporation

**Figure 2:**
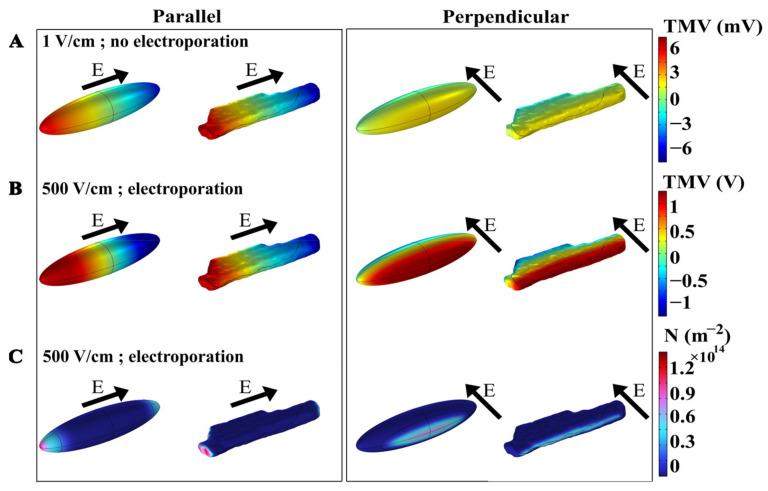
The spatial distribution of transmembrane voltage (TMV) induced by a 10 ms pulse of (**A**) 1 V/cm (without electroporation) or (**B**) 500 V/cm (with electroporation). (**C**) The spatial distribution of the pore density (m^−2^) induced by the end of a 10 ms, 500 V/cm pulse (with electroporation). In each panel, the results are shown for prolate spheroid and real-shaped geometry when the electric field is applied either parallel or perpendicular to the long axis of the cell. The direction of the applied electric field is indicated by the arrows. Note different scales of TMV for panels (**A**,**B**).

A time-dependent nonlinear numerical model was developed to investigate the phenomenon of electroporation when exposing a cardiomyocyte to a single monophasic electric pulse of different pulse durations, i.e., 10 ms, 1 ms, 100 µs, 10 µs, 1 µs, and 100 ns. Numerical simulations were performed for a prolate spheroid, which was previously used as a simplified model of cardiomyocyte geometry [24,30,42,43], and for a real-shaped cardiomyocyte geometry [30]. Figure 2A first shows the spatial distribution of the induced transmembrane voltage (TMV) at the end of exposure to a non-electroporating 10 ms, 1 V/cm pulse, when the cell is oriented with its long axis either parallel or perpendicular to the applied electric field. The induced TMV is, by absolute value, always the highest at the membrane regions facing the electrodes, both for prolate spheroid and real-shaped geometry, and both for parallel and perpendicular orientation of the cell. When applying a non-electroporating pulse, the induced TMV reaches higher values when the cell is oriented parallel with respect to the electric field, compared with perpendicular orientation. Figure 2B again shows the spatial distribution of the induced TMV, but now at the end of an electroporating 10 ms, 500 V/cm pulse. When the absolute value of the TMV becomes sufficiently high (several 100 mV), pores start forming in the membrane, and consequently, membrane conductivity increases and the induced transmembrane voltage settles to a value of approximately 1 V [37,44]. In this case, the maximum induced TMV is ~1 V both in the parallel and perpendicular orientation of the cell. However, the maximum pore density (number of pores formed per unit membrane area) is different in parallel and perpendicular orientations, as shown in Figure 2C. These results show that the induced transmembrane voltage and the number of pores formed in the membrane depend on the cell orientation and the strength of the applied electric field.

**Figure 3:**
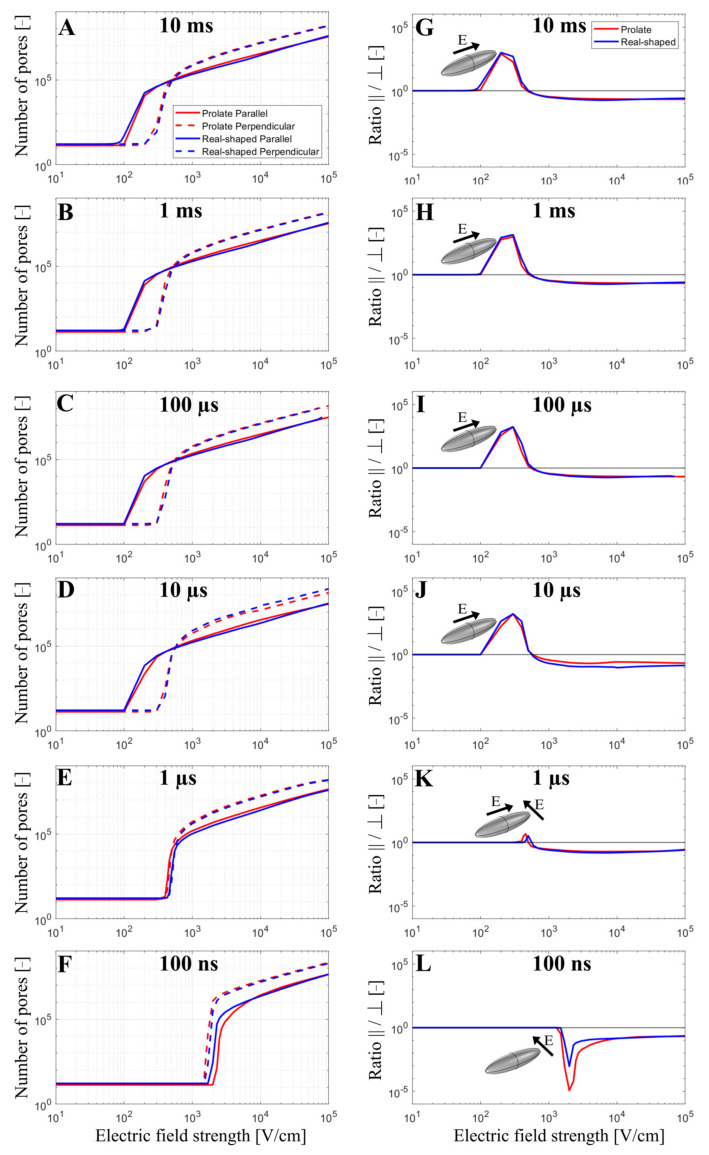
The number of pores (**A**–**F**) and the ratio of the number of pores parallel/perpendicular (**G**–**L**) as a function of the electric field when a single monophasic pulse of 10 ms, 1 ms, 100 µs, 10 µs, 1 µs, and 100 ns long is applied. In (**A**–**F**), the red and blue curves present the number of pores obtained using prolate spheroid or real-shaped geometry, respectively, when the electric field is applied parallel (solid curves) or perpendicular (dotted curves) to the long axis of the cell. In (**G**–**L**), red or blue curves represent the ratio of the number of pores parallel/perpendicular formed in the cell membrane using prolate spheroid and real-shaped geometry, respectively. The black solid line in (**G**–**L**) indicates when the ratio of the number of pores parallel/perpendicular is 1. In (**G**–**L**), the representation of the cell with the orientation of the applied electric field highlights which cell orientation is more sensitive to pore formation when the electric field is applied. The symbol [-] in the Y axis indicates that the unit of the number of pores (**A**–**F**) and the ratio parallel/perpendicular (**G**–**L**) is adimensional.

The total number of pores formed in the cell membrane was evaluated as a function of the applied electric field strength from 10 V/cm to 10^5^ V/cm when exposing the cell to a single monophasic pulse of different durations (10 ms, 1 ms, 100 µs, 10 µs, 1 µs, and 100 ns). Simulations were again performed for both prolate spheroid and real-shaped geometry and in both parallel and perpendicular orientations. The results are presented on the left side of Figure 3. Overall, the relationship between the pore number and the electric field strength for a real-shaped geometry is very similar to that of a prolate spheroid. This was additionally confirmed by comparing the local pore density in prolate spheroid and real-shaped geometry in a specified membrane region around the poles of the cell (Supplementary Figures S2 and S3). In general, the number of pores in the cell membrane always increases with increasing the applied electric field. However, the relationship between the number of pores and the applied electric field strongly depends on both the cell orientation and pulse duration. This can be observed in the graphs on the right side of Figure 3, which show the ratio of the number of pores formed in the cell membrane when the cell is oriented either parallel or perpendicular (parallel/perpendicular). For pulses with a pulse duration of ≥10 µs, cells oriented parallel to the electric field become electroporated at lower electric field strengths. In contrast, for a 1 µs pulse, the electric fields at which electroporation onsets are comparable for both perpendicular and parallel orientation. For a 100 ns pulse, cells oriented perpendicular (not parallel!) to the electric field become electroporated at lower electric field strength. Thus, there is a “crossover” at a pulse duration at the order of ~1 µs, Figure 3K, at which the orientation with the lower onset of electroporation shifts from parallel to perpendicular as observed in experiments and reported before [31].

In addition, the model suggests that the orientation, at which cells form more pores due to electroporation, depends not only on the pulse duration but also on the electric field strength. Interestingly, for pulses with a duration of ≥10 µs, parallel orientation is the one in which pores start forming preferentially (at lower electric field strengths); however, as the electric field is increased beyond a certain value, cells in perpendicular (not parallel!) orientation achieves a greater pore number and thus become more electroporated. This can be observed both for the real-shaped geometry and the prolate spheroid, suggesting that such behavior would be observed for any cell of elongated shape. Thus, the model suggests there is another “crossover” at which the orientation of cells that are more electroporated shifts from parallel to perpendicular. This crossover appears as the electric field is increased and can be observed only for pulses with a duration of ≥10 µs, Figure 3A–D. For a 1 µs pulse, the onset of electroporation occurs at similar electric field strengths for both orientations; however, the perpendicular orientation becomes more electroporated at higher electric field strengths. For a 100 ns pulse, perpendicular orientation is always more electroporated compared to parallel orientation, regardless of the applied electric field strength. 

### 3.2. The Effect of T-Tubules 

**Figure 4:**
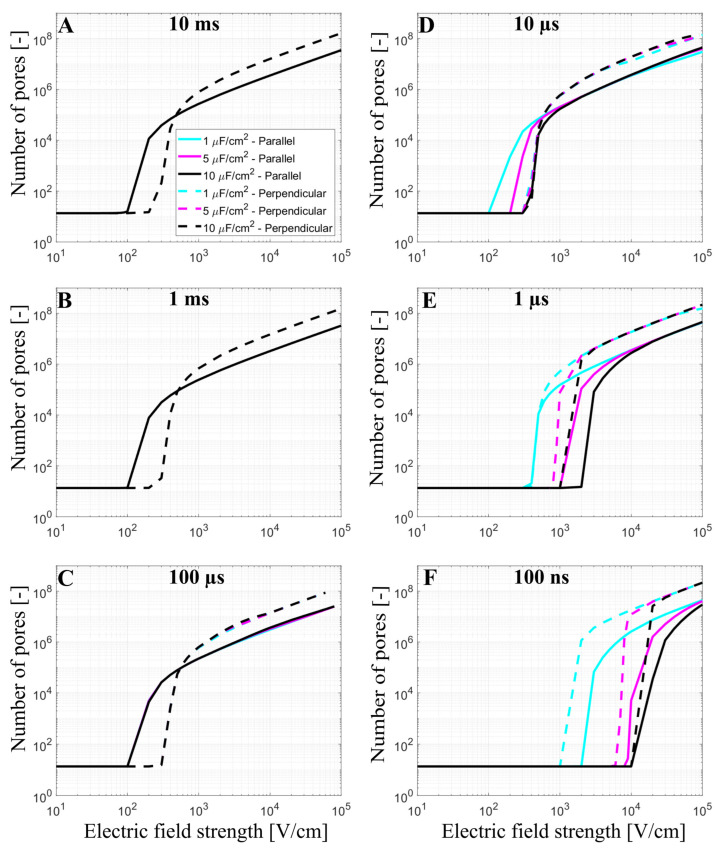
The number of pores as a function of the electric field using prolate spheroid geometry when a single monophasic pulse of (**A**) 10 ms, (**B**) 1 ms, (**C**) 100 µs, (**D**) 10 µs, (**E**) 1 µs, and (**F**) 100 ns long is applied. The electric field is applied parallel (solid line) or perpendicular (dashed line) to the long axis of the cell. The cyan, magenta, and black curves represent the number of pores obtained using a membrane capacitance of 1 µF/cm^2^, 5 µF/cm^2^, and 10 µF/m^2^, respectively. The symbol [-] in the Y axis of (**A**–**F**) indicates that the unit of the number of pores is adimensional. Please note that in (**A**–**C**) the cyan, magenta, and black lines are overlapping.

Cardiomyocytes have a complex membrane shape with many t-tubules, which results in a higher effective membrane capacitance [17,40]. T-tubules density and organization are variable depending on the type of myocyte considered. While ventricular myocytes have a dense and well-organized t-tubular network, t-tubules density is much lower in atrial cardiac myocytes or Purkinje fibers [45]. In our model, the effect of t-tubules on the formation of the pores in the membrane was considered by increasing the membrane capacitance from 1 µF/cm^2^ to 5 µF/cm^2^ and 10 µF/cm^2^. The number of pores in the cell membrane was again evaluated as a function of the applied electric field from 10 V/cm to 10^5^ V/cm at different pulse durations (10 ms, 1 ms, 100 µs, 10 µs, 1 µs, and 100 ns), Figure 4. 

The cyan, magenta, and black curves in Figure 4 represent the number of pores obtained using prolate spheroid geometry with 1 µF/cm^2^, 5 µF/cm^2^, and 10 µF/cm^2^ as membrane capacitance, respectively, when the electric field is applied parallel (solid line) or perpendicular (dashed line) to the long axis of the cell. When the membrane has a greater capacitance (representative of t-tubules), a higher electric field is needed to observe the onset of electroporation when using pulses of 100 ns, 1 µs, (for both orientations), and 10 µs (only for the parallel orientation), which is directly related to the longer membrane charging time. When 100 µs, 1 ms, and 10 ms pulses are used, the different values of capacitance do not affect the number of pores in the cell membrane, as their pulse durations are all considerably longer than the membrane charging time, even for the highest value of the capacitance. The charging time of the cardiomyocyte using the prolate spheroid geometry is ~2 µs (parallel orientation) and ~0.8 µs (perpendicular orientation). Another interesting observation is that the pulse duration, at which electroporation is not very sensitive to cell orientation (both parallel and perpendicular orientation were similarly affected), shifts from 1 µs to 10 µs, as the membrane capacitance increases from 1 µF/cm^2^ to 10 µF/cm^2^. This means that the above-mentioned “crossover” with respect to pulse duration would be observed at roughly 10× longer pulse duration in cardiomyocytes compared to other cell types with similar aspect ratios but are devoid of t-tubules.

### 3.3. Experimental Results and Model Predictions: Calcium Transients in Cardiomyocyte-Derived Cell Lines 

**Figure 5:**
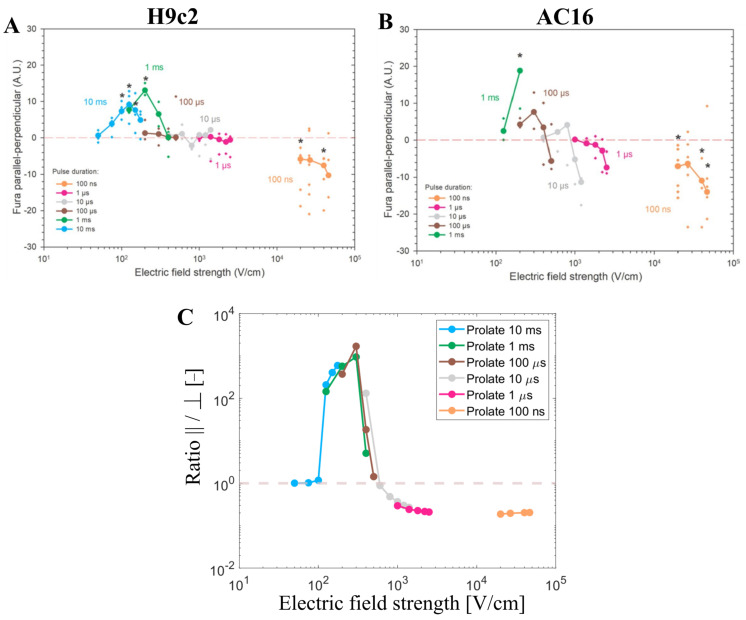
Experimental measurements of calcium transients using Fura-2 ratio 340/380 peak change using H9c2 and AC16 cell lines when different pulse durations were applied (from 10 ms down to 100 ns) published by Dermol-Černe et al. (**A**,**B**). *—statistically significant differences from control (*p* < 0.05), the Kruskal–Wallis One Way Analysis of Variance on Ranks, followed by Multiple Comparisons versus Control Group (the Dunn’s Method), see Dermol-Černe et al. [31]. Figure 5 (A,B) are reprinted with permission from [31]. (**C**) represents the ratio of the number of pores parallel/perpendicular obtained with the model using pulse durations and the electric field strengths of the one used in the experiments (**A**,**B**). The symbol [-] in the Y axis of (**C**) indicates that the unit of the ratio parallel/perpendicular is adimensional.

The numerical results that have been presented so far have shown that the total number of pores predicted by the model depends on cell orientation, pulse duration, and electric field strength, whereby the results are very similar in both prolate spheroid and real-shaped cardiomyocyte geometries. In continuation, our modeling results are compared to experimental results from cardiomyocyte-derived cell lines and primary cardiomyocytes (later in Section 3.4). Dermol-Černe et al. [31] reported experimental measurements of calcium transients using Fura-2 dye in two cardiomyocyte-derived cell lines, H9c2 and AC16, induced by monophasic pulses of different duration (from 10 ms down to 100 ns) and electric field strengths. Calcium transients were observed as a consequence of the uptake of Ca^2+^ ions to cells from the extracellular medium due to electroporation [46]. Most of the cells of both cell lines were elongated, with their long axis at least twice the size of their short one. They quantified the difference in fluorescent calcium indicator Fura-2 signal in parallel and perpendicular cells (Figure 5A,B, published data). Their study also included numerical simulations, similar to ours, for prolate spheroid cells with different aspect ratios. Consistent with their model, they observed parallel cells being electroporated at lower electric field strengths compared to perpendicular cells for long pulses (1 ms and 10 ms). For pulses of intermediate duration (1 to 100 µs), the difference in Fura-2 signal in cells parallel and perpendicular to the electric field was close to zero, meaning that cells of both orientations, parallel and perpendicular, were electroporated to a similar extent. For the shortest 100 ns pulses, cells oriented perpendicularly were the ones preferentially electroporated at lower electric field strengths. This can be observed by looking at the left-most data points of each curve in Figure 5A,B. 

Our model considering the prolate spheroid geometry was used to plot the ratio of the number of pores formed in the cell membrane when the cell is oriented either parallel or perpendicular (parallel/perpendicular) for pulse durations and the electric field strengths used in the experiments (Figure 5C). Assuming pores act as pathways for cellular calcium influx induced by electroporation, the total number of pores is expected to be roughly proportional to the maximum achievable intracellular calcium concentration. The modeling results represent well the trends observed in the aforementioned in vitro cell electroporation experiments. As was discussed extensively in the previous study [31], both experimental and modeling data show a crossover of more affected cells from parallel to perpendicular when reducing the pulse duration from milliseconds to nanoseconds. In other words, parallel cells are more sensitive for longer pulse durations (>1 µs), whereas perpendicular cells are more sensitive for shorter pulse durations (<1 µs). While the model suggests this crossover occurs around a pulse duration of ~1 µs, experimentally this crossover was observed in the range of pulse durations between 1 µs and 100 µs, whereby the shift towards shorter pulse duration in the model could be related to an underestimated membrane capacitance (representative of t-tubule presence), as shown in Section 3.2. Furthermore, our modeling results also suggest that there is another crossover from parallel to perpendicular orientation, which occurs for a given pulse duration as the electric field is increased, provided that the pulse duration is more than ~1 µs long. While this crossover has not been explicitly discussed in the previous study [31], it is evident in the experimental data, especially for AC16 cells shown in Figure 5B (see the crossing of the brown and grey lines with the horizontal line as the electric field increases). 

**Figure 6:**
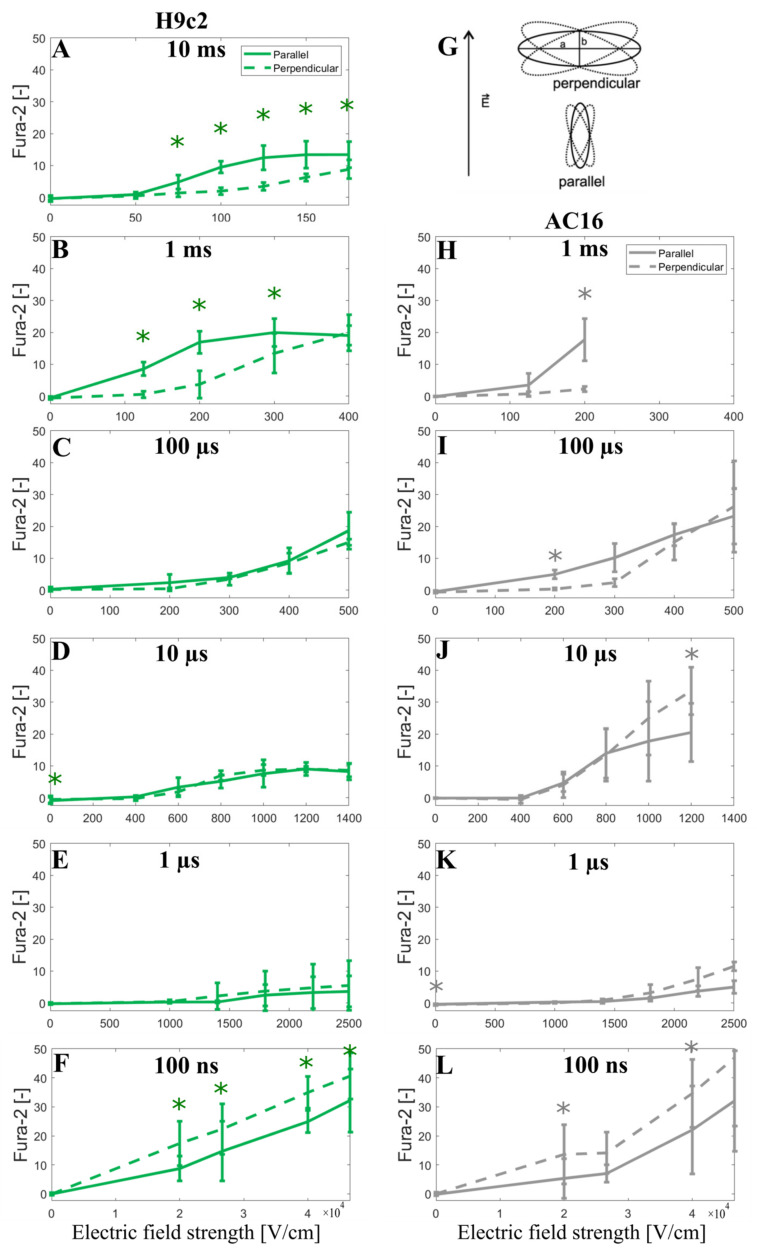
Fura-2 ratio 340/380 peak change as a function of the applied electric field using H9c2 cell line (**A**–**F**) and AC16 cell line (**H**–**L**) when a single pulse of either 10 ms, 1 ms, 100 µs, 10 µs, 1 µs, and 100 ns long is applied. (**G**) represents the elongated cells (a > 2b) with their longer axes (a) oriented parallel or perpendicular to the applied electric field E, with 20° tolerance in angle. The symbol [-] in the Y axis of (**A**–**F**,**H**–**L**) indicates that the unit of Fura-2 signal is adimensional. Experimental results of H9c2 cells (**A**–**F**) were obtained from 5–30 cells per experiment, an average of three independent experiments, except for 1 ms (N = 4), 10 ms (N = 5), 100 ns, 40 and 46.6 kV/cm (N = 5), 100 ns, 20 kV/cm (N = 6), and 100 ns, 26.6 kV/cm (N = 9). Experimental results of AC16 cells (**H**–**L**) were obtained from 4–23 cells per experiment, an average of three independent experiments, except for 100 ns, 40 kV/cm (N = 5), 100 ns, 46.6 kV/cm (N = 6), 100 ns, 20 kV/cm (N = 7) and 100 ns, 26.6 kV/cm (N = 7). N is the number of repetitions. Results are expressed as a mean ± standard deviation. *—statistically significant differences from control (*p* < 0.5), paired *t*-test.

In addition, to further support the modeling prediction of a crossover as the electric field strength is increased, we decided to show the Fura-2 ratio 340/380 peak change observed when the cells are oriented parallel or perpendicular to the applied electric field (unpublished data, see Section 2.2). Indeed, careful inspection of the results obtained with ≥1-us-long pulses shows that as the electric field increases, the Fura-2 signal becomes similar for both parallel and perpendicular cells, or even the perpendicular cells, start to exhibit a greater Fura-2 signal indicating greater calcium uptake. This was statistically significant in data for 10 μs and visible in data for 100 µs pulse in AC16 cells (Figure 6I,J). For 1 ms pulse in AC16 cells, the electric field was not increased high enough to approach this crossover; however, the crossover is indicated in the results from H9c2 where higher electric fields were used (Figure 6B). It should be noted that the Fura-2 fluorescence signal is linearly proportional to the intracellular calcium until the Fura-2 binding sites for Ca^2+^ are saturated. Unavoidably the use of high electric field strengths will induce cellular Ca^2+^ influx approaching the saturation level of the Fura-2 dye, whereby such saturation can mask the above-discussed crossover with respect to electric field strength. Thus Fura-2 calcium measurements in Figure 6 cannot unequivocally confirm the modeling predictions; nevertheless, they are not contradicting them. 

### 3.4. Experimental Results and Model Predictions: Lethal Electric Field Strengths in Primary Cardiomyocytes 

**Figure 7:**
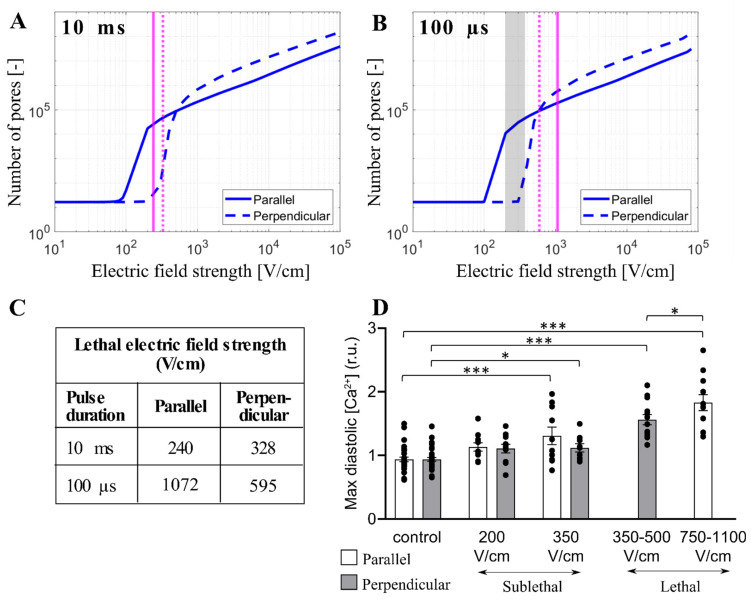
Comparison between the model and experimental results in primary cardiomyocytes. (**A**,**B**) The predicted number of pores as a function of the electric field when a single pulse of 10 ms (**A**) or 100 µs (**B**) is delivered. The electric field is applied parallel (solid blue line) or perpendicular (dashed blue line) to the long axis of the cell. The vertical solid and dashed magenta lines indicate the value of the lethal electric field for the parallel and perpendicular orientation, respectively that are reported in [33]. The symbol [-] in the Y axis of (**A**,**B**) indicates that the unit of the number of pores is adimensional. (**C**) Tabulated lethal electric field strengths were determined experimentally in [33] for parallel and perpendicular orientation when a single 10 ms or 100 µs pulse was applied. Lethal electric field strengths are estimated as the lethal voltage, reported in [33] divided by the electrode distance (4 mm). (**D**) Comparison of maximum diastolic [Ca^2+^] for parallel and perpendicular orientation after exposure to 100 µs pulse of increasing electric field strengths [33]. These sublethal electric fields (experimental results) are indicated with the grey region in (**B**). In (**D**), the results are expressed as a mean ± standard error of the mean with individual values for each cell. The asterisks represent: *: *p* < 0.05, ***: *p* < 0.001.

Monitoring intracellular calcium levels with Fura-2 can be a very sensitive indicator of electroporation at low electric field strengths [41]. However, at high electric field strengths approaching irreversible electroporation, Fura-2 binding sites can become saturated and the difference between calcium uptake in parallel and perpendicular cells becomes difficult to assess. Thus, we further compared our modeling results to the results from the study of Chaigne et al. [33] who studied electroporation in isolated primary rat cardiomyocytes. The authors showed that the lethal electric field strength depends on cardiomyocyte orientation and pulse duration. When applying a single 10 ms pulse, the lethal electric field strength for 80% probability of lethality is 240 V/cm and 328 V/cm for parallel and perpendicular orientation, respectively, meaning that cardiomyocytes oriented parallel are more sensitive to the electric field (as expected). However, when applying a single 100 µs pulse, the effect of cardiomyocyte orientation on the lethal electric field strength unexpectedly changed, and cardiomyocytes became more sensitive in perpendicular orientation; the lethal electric field strength for cardiomyocytes oriented parallel or perpendicular was found to be 1072 V/cm and 595 V/cm, respectively. We used our model considering the real-shaped cardiomyocyte geometry to help interpret the experimental results described. Our calculations (Figure 4) suggest that 10 ms, and 100 µs, pulse is too long for its effects to be influenced by increased effective membrane capacitance due to t-tubules. Thus, we plotted the relationship between the total number of pores and the electric field strength for cardiomyocytes with default membrane capacitance (1 µF/cm^2^) oriented parallel and perpendicular when exposed to a 10 ms or 100 µs pulse. Then, we indicated the lethal electric field strengths obtained in experiments, as shown in Figure 7A,B. For electric field strengths, which were lethal at a 10 ms pulse, there were considerably more pores formed in parallel orientation (Figure 7A), supporting the experimental observation that parallel cells are more sensitive than perpendicular. However, for higher electric field strengths, which are lethal at 100 µs pulse, there are more pores formed in perpendicular orientation compared to parallel orientation (and a greater fraction of the membrane area electroporated, Supplementary Figure S4), supporting the experimental observation that perpendicular cells are more prone to irreversible electroporation when exposed to 100 µs pulse. These experiments thus additionally support the modeling prediction that there is a crossover with respect to the electric field strength, at which perpendicular cells become more affected compared to parallel cells. To further confirm that there is indeed a crossover that occurs at high electric field strengths, Figure 7D presents experimental measurements of Ca^2+^ uptake using Fura-2 dye in rat primary cardiomyocytes exposed to 100 µs pulse but of lower (sublethal) electric field strengths. Otherwise, the electroporation protocol was the same as in Chaigne et al. [33]. The results in Figure 7D present the level of diastolic intracellular Ca^2+^ measured after exposure to an electric pulse. We consider that the level of diastolic Ca^2+^ can be best compared to our model, which predicts the number of pores in the membrane formed by the electric pulse. Indeed, the experiments show that the level of diastolic Ca^2+^ becomes more increased in parallel cells compared to perpendicular cells for these lower electric field strengths, consistent with the model (vertical dashed lines in Figure 7B indicate these lower electric field strengths and show that the model predicts a greater number of pores formed in parallel cells).

### 3.5. Limitations of the Model 

Our modeling results suggest that the orientation at which cardiomyocytes (and other elongated cells) become preferentially affected by electroporation pulses depends both on pulse duration and the electric field strength. According to the model, there are two crossovers were preferentially affected orientation shifts from parallel to perpendicular. One crossover can be observed for relatively low electric fields (likely corresponding to reversible electroporation, i.e., sublethal) when reducing the pulse duration from milliseconds to nanoseconds. The other crossover can be observed for pulses with a duration of >1 µs when increasing the electric field strength. The first crossover has been confirmed previously by measurements of calcium uptake into cardiomyocyte-derived cell lines [31]. The second crossover identified in this study is supported by measurements of the lethal electric field strengths and calcium uptake in primary rat cardiomyocytes ([33] and Figure 7C,D), and by the revisited measurements in cardiomyocyte-derived cell lines (Figure 6). Thus, the prediction of the two crossovers seems to be robustly confirmed by experimental data. 

Nevertheless, the existing electroporation models, including the one used in this study, have several limitations [47]. Electroporation models consider that the increase in membrane permeability can mainly be attributed to pores formed in the lipid bilayer of the cell membrane. While this mechanism is probably dominant during the on-time of the pulse, other mechanisms are considered to contribute and may even dominate in the increased membrane permeability after the pulse, including membrane defects due to oxidative lipid damage [48,49,50] and electric-field mediated perturbation of membrane proteins such as voltage-gated ion channels [51]. This post-pulse increase in permeability lasts up to several minutes (at room and physiological temperature) and mediates most of the transmembrane transport of small ions, such as calcium, and small molecules, such as ATP [52,53,54]. Furthermore, the resealing process of the cell membrane that takes place after the pulse is not merely a passive pore closure as assumed in the models, but involves membrane repair mechanisms, such as exocytosis and endocytosis [55,56].

Our electroporation model in principle considers only lipid pores that form in the cell membrane during the pulse. Nevertheless, it is reasonable to assume that other effects, including the post-pulse increased membrane permeability, intracellular calcium uptake, and effects leading to cell death, are at least roughly correlated with the number of pores that we simulate with our model. Indeed, the model predictions are found to be qualitatively in agreement with experimental measurements of calcium uptake and lethal electric field strength when assessing the orientation in which the cell is preferentially affected for a given pulse duration and electric field strength. However, the total number of pores returned by the model has limited utility for predicting lethal electric field strength (irrespective of orientation) for different pulse durations. Specifically, the model predicts a considerably greater number of pores at electric field strengths that are lethal when applying a 100 µs pulse (>10^5^ pores) compared to a 10 ms pulse (>2 × 10^3^ pores), cf. Figure 7A,B. Thus, the pore number predicted by the model cannot be used as a proxy for cell death when comparing pulses of different duration. Indeed there are additional effects associated with electroporation that could contribute to cell death, apart from pore formation and increased membrane permeability, including electrodeformation of the cell shape [57] and disassembly of the cytoskeletal network [58], both of which are expected to be more profound with longer pulses. Overall, the mechanisms of cell death following electroporation at present remain elusive and require further research [59]. 

In our model, we investigated the effects of an external electric field on a single cardiomyocyte when a single monophasic pulse of different pulse durations and electric field strengths was applied. Our modeling is largely motivated by in vitro experimental data published by Dermol-Černe et al. [31] and Chaigne et al. [33] and is aimed at gaining a better understanding thereof. However, clinical applications of PFA use a wide range of pulse waveforms, including monophasic and biphasic pulses with durations ranging from nanoseconds to milliseconds, and more importantly, most of these waveforms are composed of large numbers of pulses—trains of pulses [2,28,29]. Therefore, it is important to admit that existing electroporation models (including the one used in our study) have strong limitations when it comes to representing the experimentally observed cumulative effect of the number of pulses, as shown in ours and others’ previous numerical studies [47,60]. Thus, further development of electroporation models is required to model more complex pulse waveforms that are being used in or are being developed for clinical applications. 

While our model considered a single cardiomyocyte, our modeling findings regarding the influence of cell orientation for given pulse duration and electric field strength are nevertheless informative for interpreting the results at the tissue level. Even though in tissue, the electric field distribution is always inhomogeneous due to many factors, among them electrode/catheter geometry, tissue conductivity heterogeneity, and anisotropy, it should be noted that an individual cell within the tissue will be locally exposed to roughly homogeneous field of a specific magnitude over the length scale of one cell (similarly as modeled in our study).

Finally, in our model, we consider the effects of t-tubules only by changing the values of membrane capacitance since the explicit representation of t-tubules is computationally much more demanding due to their small and elongated size, which requires a potentially large number of discretization elements (finite elements in our case) and more importantly difficult validation of the results. However, in the future, it would be interesting to model the realistic structure of t-tubules in the cardiomyocyte studying how they affect electroporation. 

### 3.6. Clinical Relevance

Understanding the effect of orientation with regard to sensitivity to electrical fields in anisotropic tissues, such as the cardiac tissue, is important, and this particular study further informs translational research aimed at developing novel and improved PFA device therapies. One issue that has become apparent in the PFA literature is the relatively high variability in lesion size using PFA devices, a finding that merits further investigation [61,62,63,64]. Tissue anisotropy and the specific orientation of the cardiac myocytes to the electric fields on ablation electrodes are possible hypotheses for this observation. 

While the present investigation has focused on modeling ventricular cells, the primary anatomical targets for PFA are currently the pulmonary veins (PVs) to isolate PVs from the rest of the atrium. Rapid electrical activity in PVs has been suggested as a key mechanism for focal atrial fibrillation [65]. The myocardial anatomy, histology, and architecture of PVs, while similar to atrial myocardium [66], is different from ventricular cells. PV myocytes are oriented circumferentially in the veins and the PV sleeves containing the myocytes extend into the veins for 4–20 mm [66]. Atrial myocytes are generally thinner than ventricular myocytes with reported widths of 6 to 15 µm [67,68], while the length is within the range of ventricular myocytes (120 µm) [68]. Thus, the reported calculations in the present paper are relevant for not only ventricular but also atrial cells, including the atrial cells of the PVs. 

Interestingly, the orientation of the atrial myocytes in the PVs will likely be perpendicular to applied fields due to their circumferential orientation in the PV sleeves, assuming circular or balloon-shaped PFA ablation electrodes. Based on the present data, it could then be argued that a pulse duration of 100 ns might be advantageous as cells oriented perpendicular (not parallel) to the electric field become electroporated at lower electric field strengths. Thus, a relatively lower electric field strength would be needed to achieve electroporation in perpendicularly oriented PV cardiomyocytes when a 100 ns pulse was used, which could, at least in theory, further improve the safety profile of the therapy. 

Conversely, in tissues where the orientation of tissue relative to the ablation electrodes is difficult to control, a 1–10 µs pulse may be advantageous as the electric fields at which electroporation onsets are comparable for both perpendicular and parallel orientation (per Figure 3 and Figure 4), so in a sense is “orientation agnostic”, i.e., independent of orientation. For example, ventricular tissue is known to be multi-layered, and heavily trabeculated, and the orientation of the ablation electrodes (with various shapes and resulting electrical fields) relative to those tissues are less controlled and conceivably less predictable than in a simpler PV anatomy. The example does not consider the effects of t-tubules (which results in increases in membrane capacitance and a shift of those cross-over values).

It needs to be understood that the aforementioned is a little bit of an oversimplification as at higher field strengths perpendicular cardiomyocytes become more electroporated. In this respect, it should also be stressed that the electric field around the catheter will always be high and decrease with distance from the catheter, so the simultaneous presence of high and low electric fields is unavoidable. Therefore, the complex interplay between pulse durations, electric field strengths, and cell orientation should in general always be taken into account. These examples in principle demonstrate how findings from numerical models describing electroporation at the single-cell level can inform translational PFA research efforts. 

## 4. Conclusions

To study the effect of electroporation on a single cardiomyocyte we developed a time-dependent nonlinear numerical model of electroporation building upon Milan et al. model [30]. In particular, we investigated how the induced transmembrane voltage and the number of pores in the cell membrane are affected when applying a monophasic pulse of different pulse durations and electric field strength, considering different cell shapes, the presence of t-tubules by increasing the membrane capacitance, and the cell orientation with respect to the applied electric field. To gain a better understanding of seemingly conflicting in vitro experimental data published by Dermol Černe et al. [31] and Chaigne et al. [33], we compared the experimental results with the modeling findings.

The modeling results show that prolate spheroid geometry is a reasonable approximation of a real-shaped cardiomyocyte geometry when modeling electroporation. In addition, prolate spheroid geometry (simple geometry) is computationally less demanding than using real-shaped geometry (complex geometry). The main difference between both geometries is at intermediate electric field strengths, where the onset of electroporation slightly differs for a prolate spheroid and real-shaped cardiomyocyte. The presence of t-tubules effectively increases the membrane capacitance and thus affects electroporation when 100 ns, 1 µs, and 10 µs pulses are used. Thus, t-tubules should be taken into account when modeling electroporation of cardiomyocytes exposed to pulses with a duration less or equal to ~10 µs. This becomes relevant for cardiac cell types which have many t-tubules, such as ventricular cardiomyocytes being targeted in ventricular ablations, which may thus require longer pulses. 

The orientation at which the cells are preferentially affected by electroporation depends on both pulse duration and electric field strength. For sub-microsecond pulses, cells are more affected in the perpendicular orientation at all electric field strengths. For pulses with duration on the order of 1 µs, the onset of electroporation is observed at comparable electric field strengths regardless of the orientation; however, as the electric field is increased considerably beyond the onset, perpendicularly oriented cells become more affected. For pulses on the order 10 µs and longer, the onset of electroporation is observed at lower electric field strengths for parallel orientation; however, perpendicularly oriented cells become more affected as the electric field is increased. The presence of t-tubules shifts these crossovers to the right towards longer pulse durations. Thus, our modeling findings show that for low electric fields with 100 µs pulse duration, the parallel orientation is more affected than the perpendicular one according to Dermol-Černe et al. [31]. However, interestingly, for higher electric fields, there is a shift and the perpendicular orientation is more sensitive than the parallel one according to Chaigne et al. [33] with 100 µs pulse duration. Thus, in vitro experimental results from the two studies can be explained by our model. 

Our results are important for developing electroporation for cardiac treatments, including irreversible electroporation for cardiac ablation, i.e., Pulsed Field Ablation [28], and reversible electroporation for cardiac gene therapy [69]. The presented model can aid in interpreting experimental results on the influence of cell orientation on electroporation propensity.

## Figures and Tables

**Table 1:** Model parameters.

Parameter	Symbol	Value	Ref.
Intracellular permittivity	*ε_i_*	80	[39]
Extracellular permittivity	*ε_e_*	80	[39]
Intracellular conductivity	*σ_i_*	0.8 S/m	[30]
Extracellular conductivity	*σ_e_*	1.4 S/m	[30]
Membrane conductivity	*σ_m_*	1.4925 × 10^−8^ S/m	[30]
Membrane thickness	*d_m_*	5 nm	[37]
Membrane capacitance	*C_m_*	0 μF/cm^2^ 5–10 μF/cm^2^ *	[37] [40]
Block length	*L*	400 µm	Arbitrary
Electroporation constant	*q*	1.46	[31]
Electroporation parameter	*A*	10^9^ 1/(m^2^s)	[37]
Characteristic voltage of electroporation	*V_ep_*	0.258 V,	[37]
Equilibrium pore density	*N_0_*	1.5∙10^9^ 1/m^2^	[37]
Pore radius	*r_p_*	0.76 nm	[37]
Pore Conductivity (cell membrane)	*σ_p_*	(σ_e_ – σ_i_)/ln(σ_e_ – σ_i_)	[36]

* Used in calculations presented in Figure 4.

## Data Availability

The data presented in this study are available on request from the corresponding author.

## Supplementary Materials

The following supporting information can be downloaded at: https://www.mdpi.com/article/10.3390/biom13050727/s1, Figure S1: The in vitro experimental setup for calcium transients in cardiomyocyte-derived cell lines; Figure S2: Areas in which the average induced transmembrane voltage and the average pore density are evaluated; Figure S3: The pore density and the relative error of the logarithmic values of the pore density as a function of the electric field when a single pulse of 10 ms, 1 ms, 100 µs, 10 µs, 1 µs, and 100 ns is applied; Figure S4: The predicted number of pores and the area with pore density >10^13^ as a function of the electric field when a single pulse of 10 ms or 100 µs long is applied.
