# Supplementary material for: Characterization of Experimentally Observed Complex Interplay between Pulse Duration, Electrical Field Strength, and Cell Orientation on Electroporation Outcome Using a Time-Dependent Nonlinear Numerical Model
Source: Biomolecules. 2023 Apr 23;13(5):727. doi: 10.3390/biom13050727 (PMC10216437; doi:10.3390/biom13050727)
Supplement: Supplementary file 1 [file biomolecules-13-00727-s001.zip › biomolecules-2283605-supplementary.pdf]

Supplementary Material

Supplementary Figure S1

Figure S1 shows the experimental setup, the images of the cells (bright and fluorescence), and the signals analysis used for the in vitro experiments performed by Dermol-Černe et al. [31].

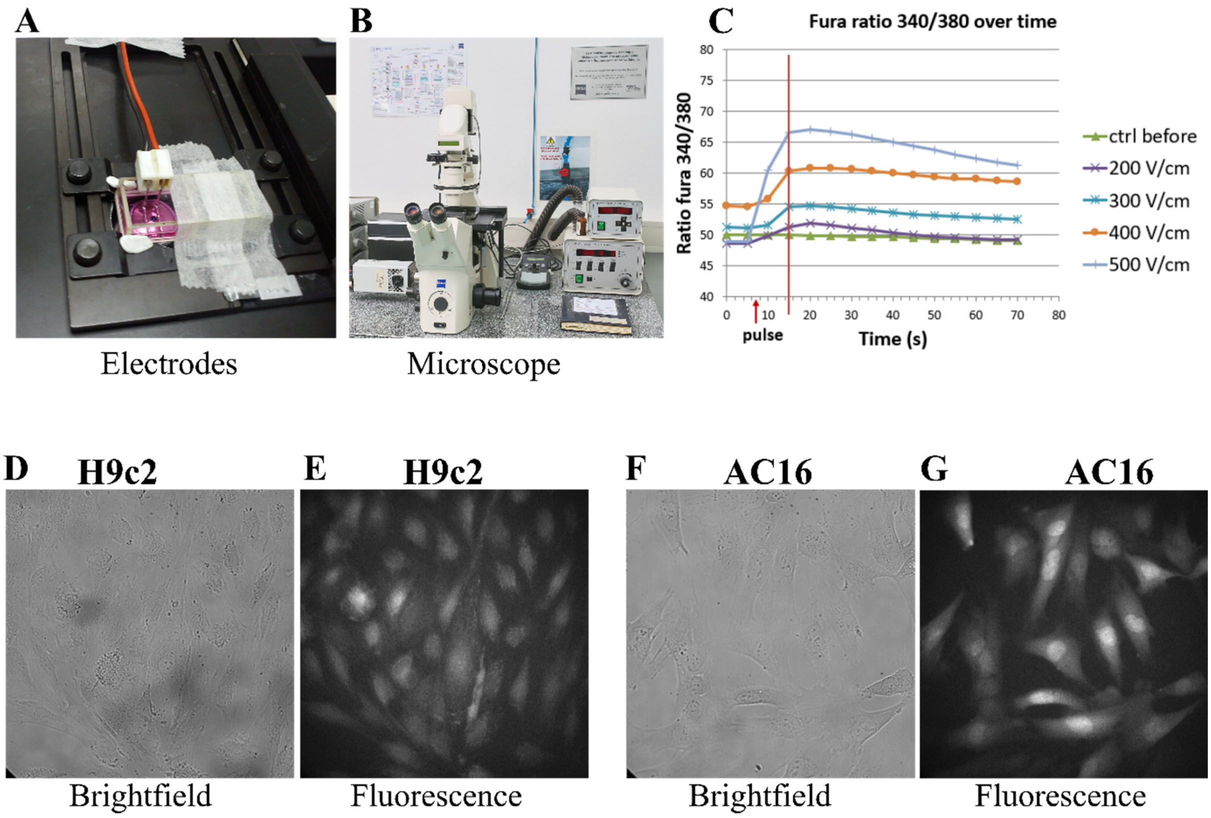

Figure S1. The *in vitro* experimental setup for calcium transients in cardiomyocyte-derived cell lines. A: The position of wire electrodes in LabTek chambers with attached cultured cells. B: An inverted fluorescence microscope (Zeiss Axiovert 200, Oberkochen, Germany). C: Experimental results of electroporation of cardiomyocytes H9c2: Fura-2 ratio 340/380 over time (results from a representative batch of cells; fura ratio was averaged from 21 cells). Experimental electroporation of H9c2 cells of different orientations were monitored by Ca^{2+} uptake with a fluorescent calcium indicator Fura-2, and image acquisition was done every 5 s. The same cells were exposed to a single pulse of 100 µs duration but with increasing voltage (200–500 V/cm) 7 s after image acquisition started (indicated with red arrow). After each pulse exposure, cells were allowed to recover. A red vertical line denotes the time of the peak fura 340/380 ratio (approximately 8 s after pulse delivery); D: Cultured H9c2 cells, brightfield; E: Cultured H9c2 cells, fluorescence (Fura-2, excitation wavelength 380 nm); F: Cultured AC16 cells, brightfield; G: Cultured AC16 cells, fluorescence (Fura-2, excitation wavelength 380 nm).

Supplementary Figures S2 and S3

The pore density in the cell membrane was evaluated in predefined membrane regions (Suppl. Fig. S2) as a function of the applied electric field from 10 V/cm to 10^5 V/cm at different pulse durations (10 ms, 1 ms, 100 µs, 10 µs, 1µs, and 100 ns) (Suppl. Fig. S3, left). To establish if the prolate spheroid geometry is a good approximation of a cardiomyocyte, we evaluated

the relative error of the difference in the logarithmic values of the pore density in the prolate spheroid and real-shaped geometry (the reference value) as a function of the applied electric field:

$$Relative\ error = \frac{|ln\ (v_{prolate\ spheroid}) - ln\ (v_{real})|}{ln\ (v_{real})}\ x\ 100\%$$

where $v_{prolate\ spheroid}$ is the pore density using prolate spheroid geometry, and v_{real} is the reference pore density using real-shaped geometry.

The calculated relative error is shown in Suppl. Fig. S3 on the right. The relative error is in general small (below ~3%), except for a narrow interval of electric field strengths, where the pores just start forming with pore density between 10^9 and 10^{10} pores/m^2. This means that the onset electric field at which electroporation is experimentally observed could be slightly different for a spheroidal cell compared to a real-shaped cardiomyocyte. However, overall, the relationship between the pore density and the electric field strength for a cardiomyocyte is very similar to that of a prolate spheroid.

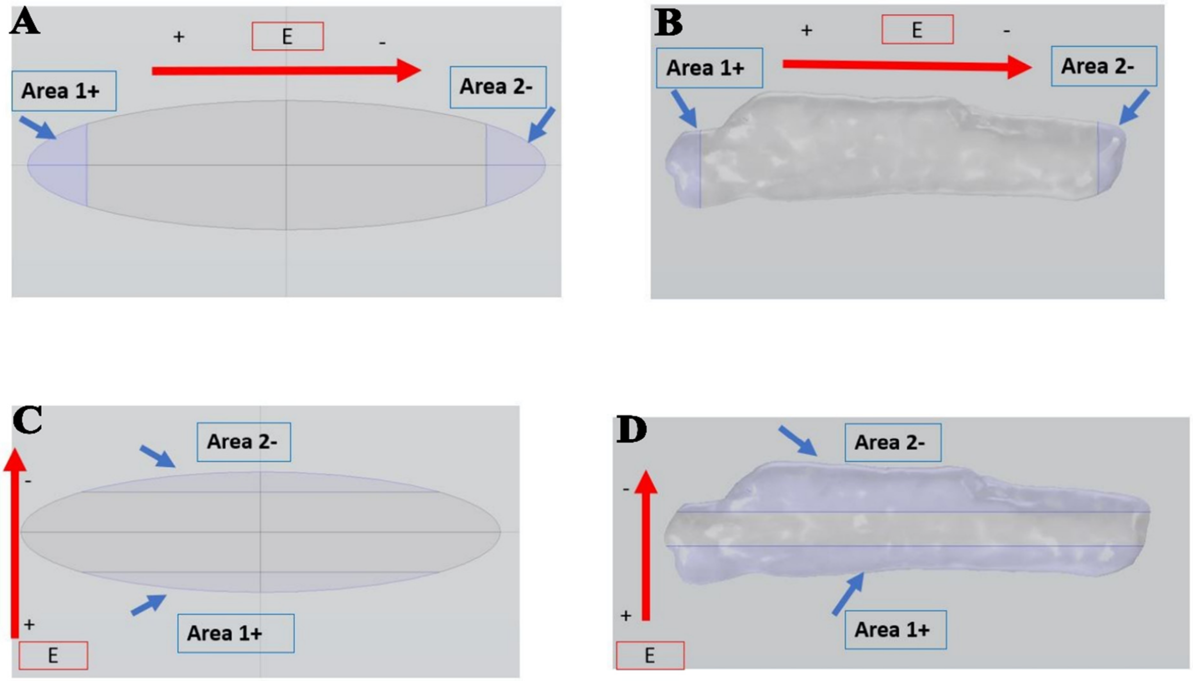

Figure S2. Highlighted in blue are the areas in which the average induced TMV and the average pore density are evaluated when the electric field is applied parallel (A–B) or perpendicular (C–D) to the long axis of the cells, respectively.

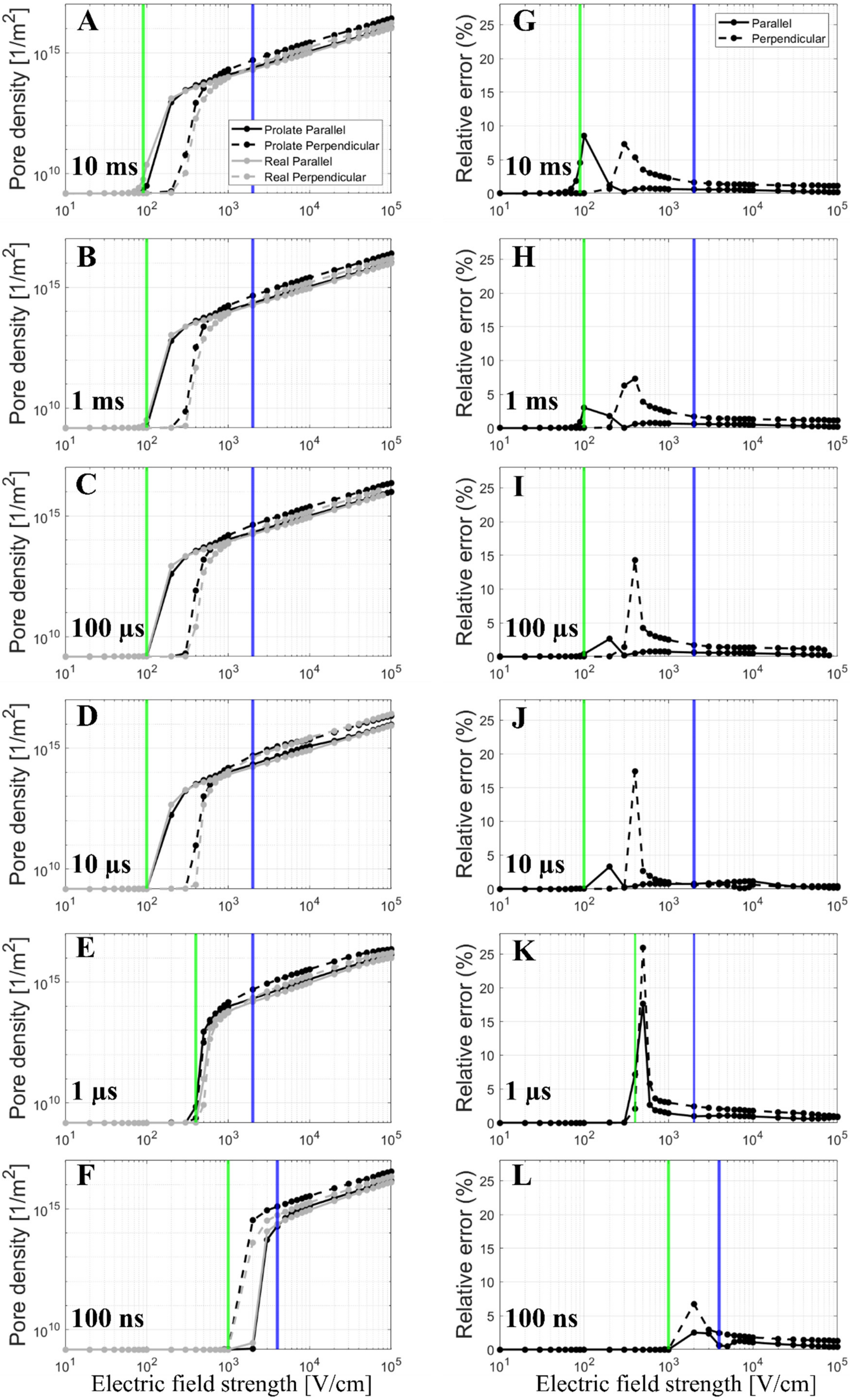

Pore density [1/m2]
Relative error (%)
Electric field strength [V/cm]
Prolate Parallel
Prolate Perpendicular
Real Parallel
Real Perpendicular
Parallel
Perpendicular
A
10 ms
B
1 ms
C
100 µs
D
10 µs
E
1 µs
F
100 ns
G
10 ms
H
1 ms
I
100 µs
J
10 µs
K
1 µs
L
100 ns

Figure S3. The pore density as a function of the electric field when a single pulse of 10 ms, 1 ms, 100 µs, 10 µs, 1 µs, and 100 ns is applied (A–F); and the relative error of the logarithmic values of the pore density (G–L). The black and gray curves represent the pore density values obtained with a prolate spheroid and real-shaped geometry, respectively. The green and blue vertical lines indicate the value of the electric field for which the pore density is below 10^{10} pores/m^2 (likely before electroporation is detected) and above 10^{14} pores/m^2 (likely when most of the cells are irreversibly electroporated), respectively.

Supplementary Figure S4

In Suppl. Fig. S4, the predicted total number of pores and the area (µm^2) of the cell in which the pore density exceeds 10^{13} is evaluated as a function of the applied electric field from 10 V/cm to 10^5 V/cm. When applying a single 10 ms pulse, the area (µm^2) of the cell in which the pore density is >10^{13} is larger in parallel orientation than in perpendicular orientation, meaning that the parallel orientation is more sensitive than the perpendicular orientation in the range of 240 V/cm to 328 V/cm electric fields, corroborating the findings from [33]. Conversely, when applying a single 100 µs pulse, the area (µm^2) of the cell in which the pore density is >10^{13} is larger for the perpendicular orientation than the parallel orientation, meaning that the perpendicular orientation is more sensitive in the range of 595 V/cm to 1072 V/cm electric fields. The grey regions indicate the value of sublethal electric fields in the range of 200 V/cm to 350 V/cm in which the parallel orientation is more sensitive than the perpendicular, corroborating the findings from [33].

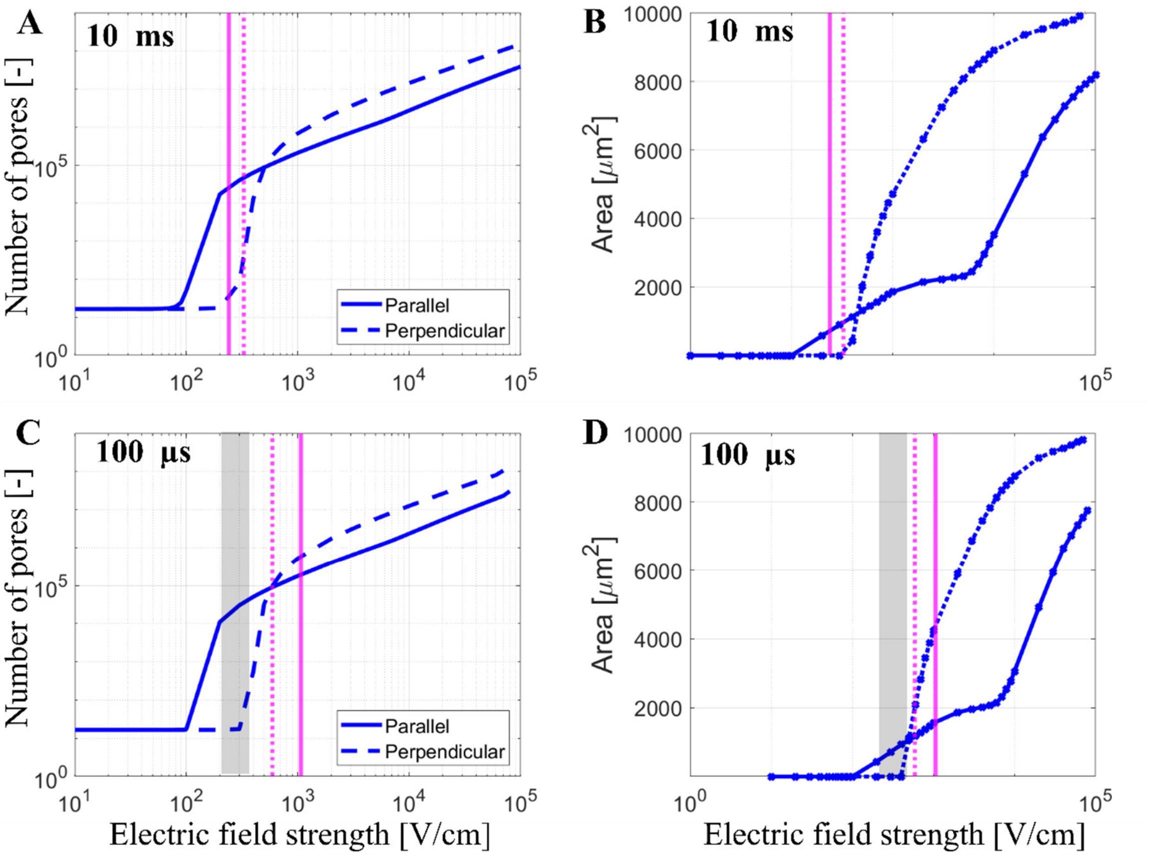

Figure S4. The predicted number of pores and the area with pore density >10^{13} as a function of the electric field when a single pulse of 10 ms (A–B) or 100 µs (C–D) long is applied. The electric field is applied parallel (solid blue line) or perpendicular (dashed blue line) to the long axis of the cell. The vertical solid and dashed magenta lines

indicate the value of the lethal electric field for the parallel and perpendicular orientation, respectively, that are reported in [33]. Sublethal electric fields (experimental results) are indicated with the grey region in Fig. C–D.
